# Fibroblast growth factor receptor 1 gene amplification and protein expression in human lung cancer

**DOI:** 10.1002/cam4.2994

**Published:** 2020-03-24

**Authors:** Omar Elakad, Anna‐Maria Lois, Katja Schmitz, Sha Yao, Sara Hugo, Laura Lukat, Marc Hinterthaner, Bernhard C. Danner, Alexander von Hammerstein‐Equord, Kirsten Reuter‐Jessen, Hans‐Ulrich Schildhaus, Philipp Ströbel, Hanibal Bohnenberger

**Affiliations:** ^1^ Institute of Pathology University Medical Center Göttingen Germany; ^2^ Department of Thoracic and Cardiovascular Surgery University Medical Center Göttingen Germany; ^3^ Present address: Innpath GmbH Innsbruck Austria; ^4^ Present address: Institute of Pathology University Hospital of Essen University of Duisburg‐Essen Essen Germany

**Keywords:** CRISPR/Cas9, *FGFR1*, fluorescence in situ hybridization, immunohistochemistry, lung cancer

## Abstract

**Background:**

Targeting fibroblast growth factor receptor 1 (*FGFR1*) is a potential treatment for squamous cell lung cancer (SQCLC). So far, treatment decision in clinical studies is based on gene amplification. However, only a minority of patients have shown durable response. Furthermore, former studies have revealed contrasting results regarding the impact of *FGFR1* amplification and expression on patient's prognosis.

**Aims:**

Here, we analyzed prevalence and correlation of *FGFR1* gene amplification and protein expression in human lung cancer and their impact on overall survival.

**Materials & Methods:**

*FGFR1* gene amplification and protein expression were analyzed by fluorescence in situ hybridization and immunohistochemistry (IHC) in 208 SQCLC and 45 small cell lung cancers (SCLC). Furthermore, FGFR1 protein expression was analyzed in 121 pulmonary adenocarcinomas (ACs). Amplification and expression were correlated to each other, clinicopathological characteristics, and overall survival.

**Results:**

*FGFR1* was amplified in 23% of SQCLC and 8% of SCLC. Amplification was correlated to males (*P* = .027) but not to overall survival. Specificity of immunostaining was verified by cellular CRISPR/Cas9 *FGFR1* knockout. FGFR1 was strongly expressed in 9% of SQCLC, 35% of AC, and 4% of SCLC. Expression was correlated to females (*P* = .0187) and to the absence of lymph node metastasis in SQCLC (*P* = .018) with no significant correlation to overall survival. Interestingly, no significant correlation between amplification and expression was detected.

**Discussion:**

*FGFR1* gene amplification does not seem to correlate to protein expression.

**Conclusion:**

We believe that patient selection for *FGFR1* inhibitors in clinical studies should be reconsidered. Neither *FGFR1* amplification nor expression influences patient's prognosis.

## Graphical abstract

Fibroblast growh factor receptor 1 (*FGFR1*) is considered a potential molecular target in squamous cell lung cancer. However, prevalence of gene amplification as well as correlation to protein overexpression have to be established. Our work has evaluated prevalence and correlation of FGFR1 gene amplification and protein expression in 421 lung cancer patient samples.

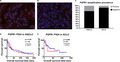

## 1 INTRODUCTION

Lung cancer is the leading cause of cancer‐related deaths in the United States and worldwide. In 2018, lung cancer occurred in approximately 2.1 million patients causing 1.8 million deaths worldwide.^1^ Lung cancer is histologically classified into small cell lung cancer (SCLC) and non‐small cell lung cancer (NSCLC). NSCLC is further classified into squamous cell lung cancer (SQCLC) and adenocarcinoma (AC). About 30% of newly diagnosed lung cancers are SQCLC.^2^, ^3^

Lung cancer has a devastating prognosis with less than 20% 5‐year survival.^4^ While early stages can potentially be cured through surgical intervention, late stages require systemic treatment like chemotherapy. However, conventional chemotherapy has demonstrated low effectiveness especially against metastasis. Targeting activating mutations like EGFR and ALK kinases in AC patients has significantly improved prognosis of these subgroups.^5^, ^6^ Unfortunately, similar specific targets are still missing in SQCLC. Recent studies have shown that fibroblast growth factor receptor 1 (*FGFR1*) gene amplification presents a potential new molecular target for SQCLC.^2^, ^7^

*FGFR1* is a member of growth factor receptor tyrosine kinases family (RTK) consisting of four receptors and 18 ligands. Receptors consist of extracellular, transmembrane, and intracellular domains.^8^ *FGFR1* play key roles in proliferation, differentiation, and migration in healthy cells through MAPK/ERK, PI3K/AKT, and JAK‐STAT pathways.^9^, ^10^ Malfunction of *FGFR1* signaling usually result from point mutations, gene amplification, or fusions.^9^, ^11^ Over‐activation of *FGFR1* signaling is tumorigenic via promoting proliferation, angiogenesis, and antiapoptotic roles in blood, bladder, gastric, breast, and prostate cancers.^9^, ^12^, ^13^, ^14^, ^15^

*FGFR1* gene amplification is one of the most frequent occurring potentially targetable gene alterations in SQCLC and SCLC with prevalence of 20% and 6%, respectively.^7^, ^16^, ^17^, ^18^, ^19^ Earlier studies have revealed comparable prevalence but inconclusive impact of *FGFR1* amplification on patients' survival.^20^, ^21^, ^22^, ^23^ Furthermore, recent clinical studies have used *FGFR1* kinase inhibitors in *FGFR1*‐amplified lung cancer patients diagnosed by fluorescence in situ hybridization analysis (FISH). However, only a small group of patients had sustainable benefit.^24^, ^25^

In this study, we aimed to study the correlation between *FGFR1* gene amplification, protein expression, clinicopathological characteristics, and prognosis in 208 SQCLC, 121 AC, and 45 SCLC patients.

## 2 METHODS

### 2.1 Tissue samples

Tissue samples were obtained from surgical resections at the department of Thoracic Surgery at the University Medical Center Goettingen, Germany. Local Ethics Committee has approved the usage of patients' materials (#1‐2‐08). All patients approved informed consent. Samples acquirement, experiments, and all procedures were held according to the Declaration of Helsinki and institutional, state, and federal guidelines.

### 2.2 Fluorescence in situ hybridization

Tissue samples were assembled in tissue microarrays, stained, and scored for *FGFR1* amplification as described previously.^26^, ^27^ Sections of 3‐4 µm thickness were mounted on slides and hybridized with ZytoLight SPEC *FGFR1*/CEN 8 (ZytoVision). VP2000 processor system (Abbott Molecular) was used for deparaffinization and protease treatment. Slides were denatured at 75°C, hybridized at 37°C, washed at 72°C, and DAPI stained. Random areas were used to count signals if *FGFR1* signal was homogeneously distributed. Sixty nuclei were analyzed for green *FGFR1* signals and orange centromere 8 (CEN8) signals. *FGFR1*/CEN8 ratio and average *FGFR1* copies/cell were calculated. Samples were considered amplified if *FGFR1*/CEN8 ≥ 2 or average count of *FGFR1* gene signals per cancer cell nucleus ≥ 6, or cancer cells which possess 15 or more *FGFR1* signals per nucleus ≥10%.

### 2.3 Cell culture

The SQCLC cell lines NCI‐H1703 and LK‐2 were purchased from AddexBio and JCRB, respectively. HCC‐15, NCI‐H2170, and NCI‐H520 cell lines were purchased from ATCC. Cell lines were kept in culture using RPMI‐1640 growth medium with 10% FCS, 1% glutamine, and 1% Penicillin‐Streptomycin. Cell lines were incubated at 37°C with 5% CO_2_.

### 2.4 Immunoblotting

Cells were lysed in RIPA buffer and loaded on 4%‐15% SDS gel (Bio‐Rad Laboratories, Inc). Gels were blotted on nitrocellulose membranes (Trans‐Blot Bio‐Rad Laboratories, Inc) and then incubated with anti‐FGFR1 antibody (D8E4, #9740, Cell Signaling). Signals were developed using Western Plus‐ECL (PerkinElmer). Expression of PARK7 (ab18257, Abcam) was used as a loading control.^28^

### 2.5 CRISPR/Cas9 design and cloning

Two gRNAs were designed to target introns before and after exon number 14 of *FGFR1* gene (5′TTCCCAGGTCCCCTAAGAGG3′ & 5′GGAGCACCAGTGTAGCCAGG3′). The gRNAs were cloned into two Cas9 plasmid backbones with green and mCherry selective fluorescence colors (PX458 and 64 217 plasmids, Addgene). H1703 cell line was transfected with both plasmids and then selected for successful transfection and sorted into single cells. Knockout was validated using immunoblotting and DNA sequencing.

### 2.6 Cell block

Cell blocks were prepared as published previously.^29^ Briefly, cell lines were harvested at 80% confluency and centrifuged. Pellets were resuspended in 500‐µL histogel (Thermo Fisher Scientific). Gels were centrifuged, chilled at 4°C for 20 minutes, and fixed in 4% buffered paraformaldehyde overnight.

### 2.7 Immunohistochemical staining

Tissue samples were assembled in tissue microarrays and stained as published previously.^30^ Shortly, tissues were cut into 2‐μm sections, then incubated in EnVision Flex Target Retrieval Solution, pH low (Dako), and then with primary antibody against *FGFR1* (dilution: 1:5000, #10646 Abcam) at room temperature for 20 minutes. Afterward, sections were incubated with secondary antibody (EnVision Flex+, Dako) and immunostaining was visualized through DAB (Dako). Mayer's hematoxylin stain was used as a counterstain. Samples were evaluated under light microscopy and staged into three stages according to intensity: (zero = negative staining; one = weak staining intensity; two = strong staining intensity). Cutoff values of FGFR1 expression were used as described previously in literature.^31^, ^32^

### 2.8 Statistical analysis

Contingency tables, chi‐square tests, and Pearson's coefficient were used to analyze correlation between patients' pathological features and *FGFR1* amplification or expression. Kaplan‐Meier analysis was used to correlate patients' survival to either *FGFR1* amplification or expression. *P*‐values were calculated according to Mantel‐Cox chi‐square test using GraphPad Prism 7. Statistical significance of *P*‐values was suggested at *P* < .05.

## 3 RESULTS

### 3.1 Clinical characteristics of patients

A total number of 421 lung cancer patient samples were collected to study gene amplification and protein expression of *FGFR1* in human lung cancer. Histopathological and clinical features are summarized in Table 1 and detailed in Table S1. Two hundred and eight patients (49.4%) were diagnosed with SQCLC, 121 patients (28.7%) with AC, and 45 patients (10.7%) with SCLC. The collective consisted of 70.1% males and 29.9% females with median age of 66 (range = 34‐85).

**TABLE 1:** Patient characteristics

Feature	Cases	SQCLC	AC	SCLC
Total	374	121	208	45
Age, median (range)	66 (34‐85)	67 (34‐85)	66 (42‐83)	67 (34‐81)
Gender				
Male	277 (74.1%)	67 (24.2%)	177 (63.9%)	33 (11.9%)
Female	97 (25.9%)	54 (55.7%)	31 (31.9%)	12 (12.4%)
Degree of differentiation				
II	249 (66.6%)	89 (35.7%)	160 (64.3%)	0 (0%)
III	125 (33.4%)	32 (25.6%)	48 (38.4%)	45 (36%)
Lymph node metastasis				
Yes	139 (39.5%)	42 (30.2%)	85 (61.2%)	12 (8.6%)
No	213 (60.5%)	70 (32.8%)	119 (55.9%)	24 (11.3%)
Clinical stage				
I+II	270 (75.8%)	92 (34.0%)	150 (55.6%)	28 (10.4%)
III+IV	86 (24.2%)	26 (30.2%)	57 (66.3%)	3 (3.5%)
Resection status				
R0	330 (90.4%)	109 (33.0%)	188 (57.0%)	33 (10.0%)
R1+2	35 (9.6%)	8 (22.9%)	20 (57.1%)	7 (20.0%)

Abbreviations: AC, adenocarcinoma; SCLC, small cell lung cancer; SQCLC, squamous cell lung cancer.

Patients were treated with surgical resection at different tumor stages (UICC, 7th edition): 47.7% were in stage I followed by 25.9% in stage II, 19% in stage III, and 1.9% in stage IV. Median follow‐up time was 30 months (1‐196 months). During this period, 227 patients deceased while 166 were alive.

### 3.2 *FGFR1* amplification in SQCLC and SCLC

Squamous cell lung cancer and SCLC samples were hybridized with fluorescent probes against *FGFR1* gene on chromosome 8 and amplification status was evaluated as described in materials and methods. In order to check for amplification heterogeneity, we counted *FGFR1*/CEN8 signals in 60 nuclei from separated regions on full slides of eight patients. *FGFR1* copy number variances (0.001, 0.007, 0.1, 0.14, 0.46, 0.63, and 1.39) indicated that at least in these samples there was no strong heterogeneity (Table S2).

**FIGURE 1:**
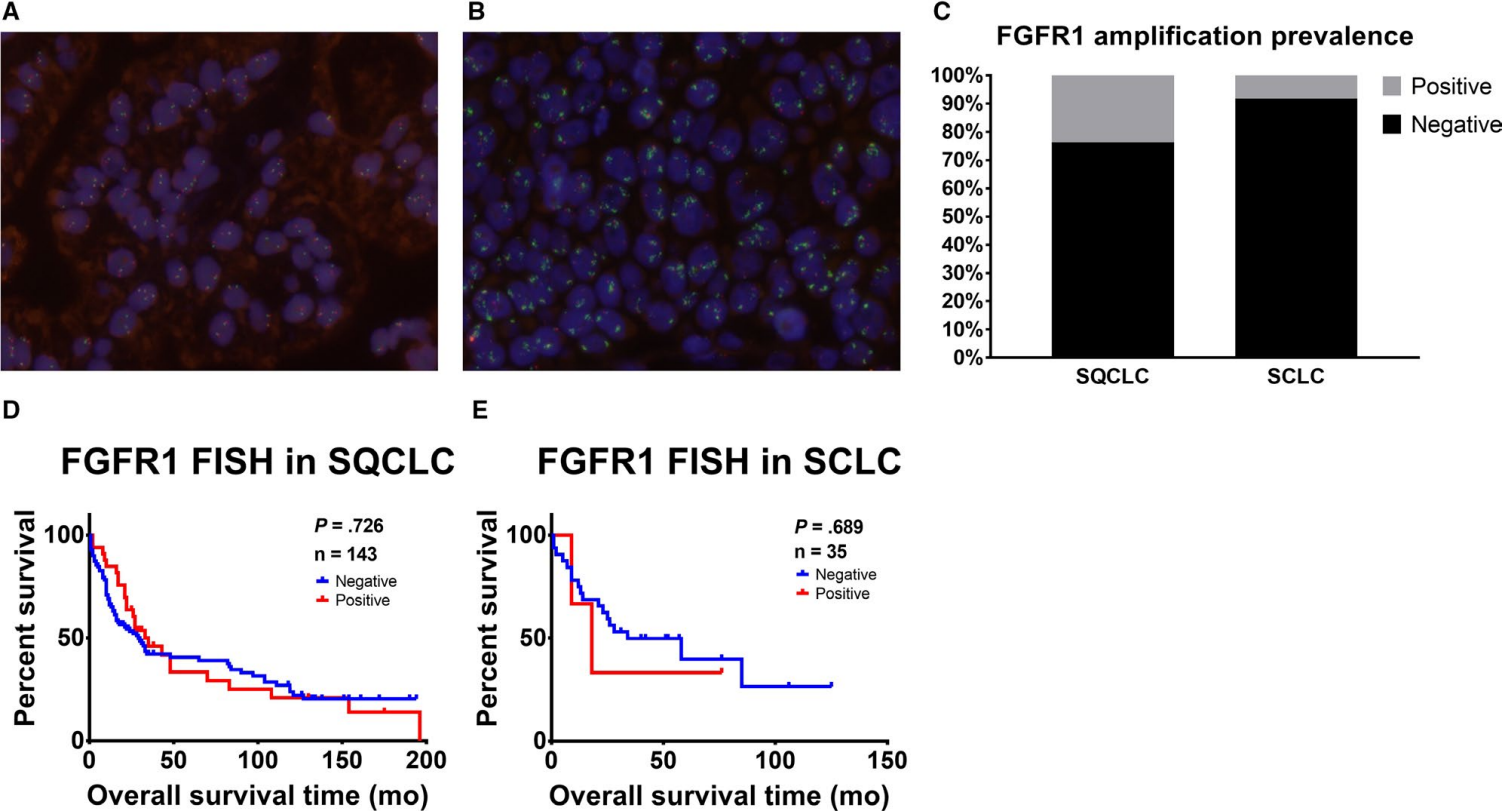
*FGFR1* gene amplification in human lung cancer. FISH analysis of *FGFR1* gene amplification showing an amplified sample (A) and a non‐amplified sample (B). Prevalence of *FGFR1* gene amplification in squamous cell lung cancer and small cell lung cancer (C). Kaplan‐Meier survival analysis according to *FGFR1* gene amplification in squamous cell lung cancer (D) and small cell lung cancer patients (E). *P*‐values were calculated according to Mantel‐Cox chi‐square test

Thirty‐seven of 156 (23%) evaluable SQCLC samples and 3 of 37 (8%) evaluable SCLC samples were amplified (Figure 1A‐C).

Median survival time in SQCLC was 27 and 19.5 months in amplified and non‐amplified groups, respectively (*P* = .73, HR = 0.92, 95% CI = 0.58‐1.45) (Figure 1D). In SCLC, median survival was 18 and 32.5 months in amplified and non‐amplified groups, respectively (*P* = .69, HR = 1.4, 95% CI = 0.26‐7.51) (Figure 1E).

### 3.3 Validation of immunostaining

**FIGURE 2:**
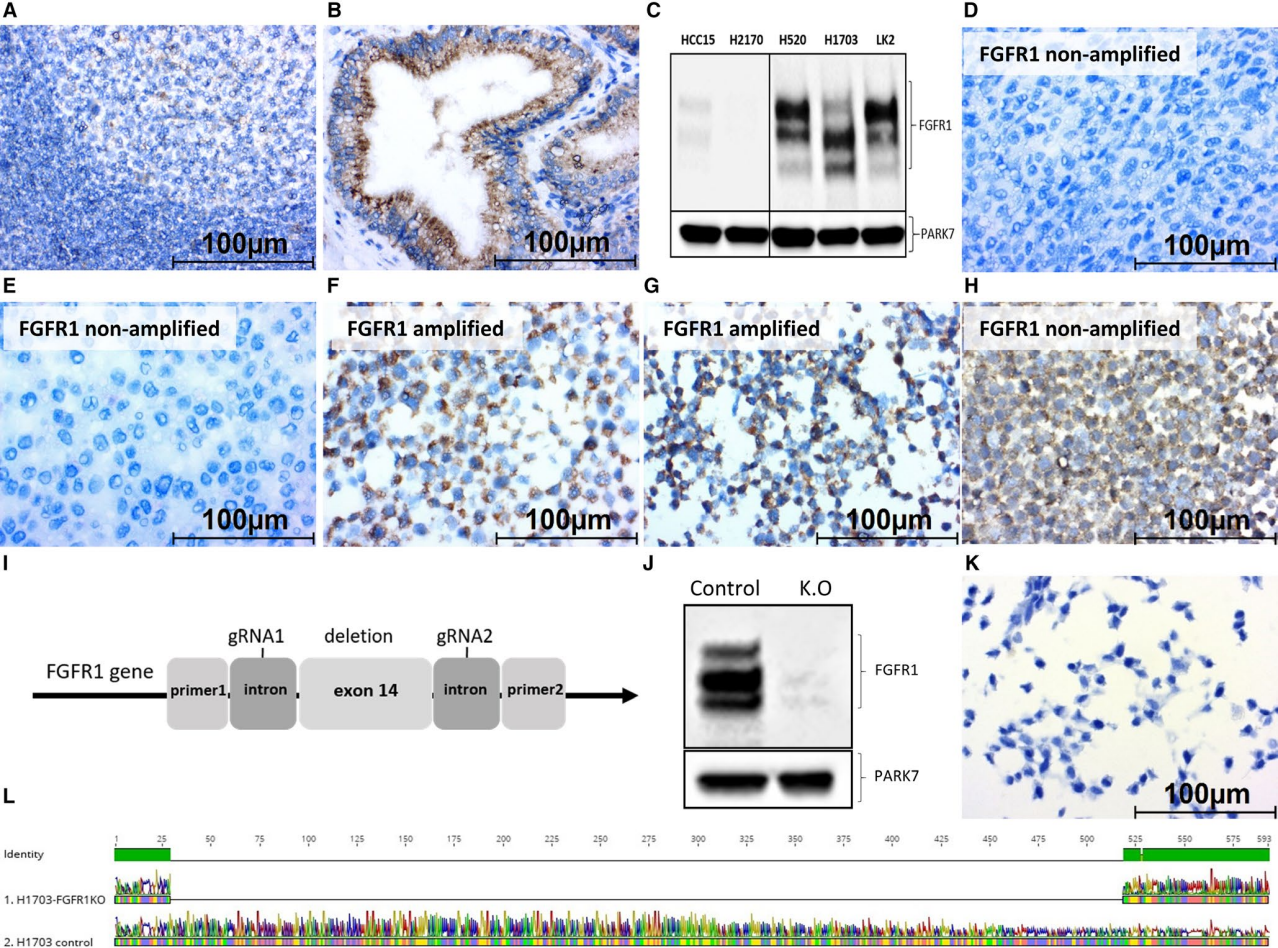
Validation of FGFR1 immunostaining antibody. FGFR1 protein expression in tonsil (A) and gallbladder (B) tissues. Immunoblotting showing FGFR1 expression in different SQCLC cell lines (C), PARK7 is used as loading control. Immunocytochemistry staining of FGFR1 in low expressing non‐amplified cell lines HCC15 (D) and H2170 (E), high expressing amplified cell lines H520 (F) and H1703 (G), and high expressing non‐amplified cell line LK‐2 (H). All images were captured at 40x magnification. Diagram showing strategy of *FGFR1* gene knockout by CRISPR/Cas9 (I). Immunoblotting of parental H1703 and H1703‐*FGFR1*‐knockout (J). Immunocytochemistry of H1703‐*FGFR1*‐knockout stained for FGFR1 (K). DNA sequencing of *FGFR1* exon 14 in H1703 control and knockout cell lines (L)

Immunohistochemistry (IHC) can lead to false positive/negative results due to off‐target binding. Three validation methods were used to confirm specificity of immunostaining. Firstly, we stained tonsil and gallbladder tissue samples and could confirm weak and strong expression, respectively, as described by various protein and RNA expression databases (Figure 2A,B).^33^, ^34^ Secondly, we stained five SQCLC cell lines and again could confirm strong expression in H520, H1703, and LK‐2 and minimal expression in H2170 and HCC15 by western blotting and immunocytochemistry (ICC) as described previously (Figure 2C‐H).^35^, ^36^, ^37^ Finally, we knocked out *FGFR1* gene in H1703 cell line through targeting exon number 14 using CRISPR/Cas9 system. Exon 14 (191 nucleotides, not divisible by three) was completely deleted by targeting introns upstream and downstream causing a frameshift mutation (Figure 2I). Exon 14 is a part of the tyrosine kinase domain and is common in all 21 *FGFR1* isoforms. Knockout was confirmed by western blotting and DNA sequencing (Figure 2J,L). Likewise, immunocytochemistry of the knockout cell line showed no expression signal confirming specificity of the antibody (Figure 2K).

### 3.4 FGFR1 protein expression in lung cancer

**FIGURE 3:**
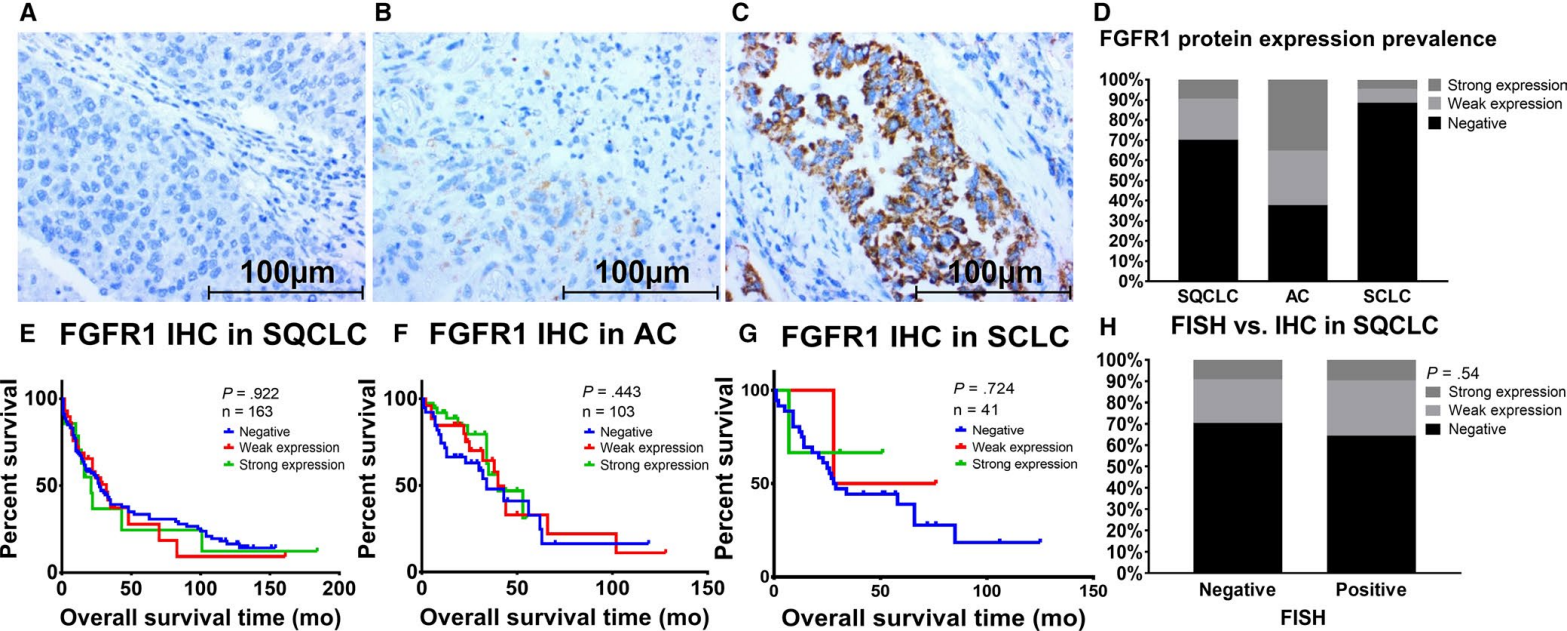
FGFR1 protein expression in human lung cancer. Negative (A), weak (B), and strong (C) cytoplasmic/membranous staining of FGFR1 in SQCLC samples. All images were captured at 40× magnification. Prevalence of FGFR1 protein expression in SQCLC, AC, and SCLC patients (D). Kaplan‐Meier survival analysis according to FGFR1 protein expression levels in SQCLC (E), AC (F), and SCLC patients (G). Correlation between *FGFR1* gene amplification and protein expression in SQCLC samples. *P*‐values were calculated according to chi‐square test (H)

Tissue sections were stained with the described antibody against FGFR1 and a homogenous staining pattern was observed (Figure 3A‐C; Figure S1). Nine percent of SQCLC (16 of 171 samples), 35% of AC (40 of 114 samples), and 4% of SCLC (2 of 44 samples) showed strong expression (Figure 3D). *FGFR1* expression had no significant impact on overall survival (*P* = .92, .44, and .72 in SQCLC, AC, and SCLC, respectively) (Figure 3E‐G). Median survival of patients with negative, weak, and strong staining was 23.5, 26, and 16.5 months in SQCLC, 21, 28.5, and 19 months in AC, and 28.5, 31, and 52 months in SCLC, respectively.

### 3.5 Correlation between *FGFR1* gene amplification and protein expression

In clinical studies, patient selection for *FGFR1‐*targeted therapy is based on gene amplification diagnosed by FISH analysis. Surprisingly, we found that *FGFR1* gene expression does not correlate to its amplification status. In SQCLC, we found positive expression in 35.5% (n = 11) of amplified and 29.6% (n = 29) of non‐amplified cases (*P* = .54, Figure 3H). Correlating patients' overall survival between these groups showed nonsignificant correlation (*P* = .95, Figure S2). In SCLC samples, all three amplified samples showed no protein expression (*P* = .99, Figure S3). Furthermore, LK‐2 (SQCLC cell line) showed strong protein expression without amplification of *FGFR1* (Figure 2H).

Amplification and expression of *FGFR1* correlated contradictory on gender. On the one hand, *FGFR1* amplification was significantly higher in males (*P* = .027), while *FGFR1* expression was significantly higher in females (*P* = .019) (Table 2). The same (albeit not significant) trend was found in SQCLC and SCLC groups when they were analyzed separately (Table 3; Table S4). Furthermore, both amplification and expression of *FGFR1* were significantly associated with better degree of differentiation (*P* = .014 and .001, respectively) (Table 2). In SQCLC, FGFR1 protein expression was significantly associated with negative lymph node status and consequently more common in UICC stage I and II (*P* = .019 and .017, respectively) (Table 3). No further significant associations between *FGFR1* amplification, protein expression, and clinicopathological characteristics were found.

**TABLE 2:** FGFR1 amplification and protein expression in SQCLC, AC, and SCLC patient samples

Feature	FISH				IHC			
	Cases	−	+	*P*	Cases	−	+	*P*
Gender								
Male	161 (83.4%)	123 (76.4%)	38 (23.6%)	.027*	239 (72.6%)	156 (65.3%)	83 (34.7%)	.0187*
Female	32 (16.6%)	30 (93.8%)	2 (6.3%)		90 (27.4%)	46 (51.1%)	44 (48.9%)	
Age								
≥60	149 (77.2%)	115 (77.2%)	34 (22.8%)	.1867^ns^	246 (75.0%)	147 (59.8%)	99 (40.2%)	.3263^ns^
<60	44 (22.8%)	38 (86.4%)	6 (13.6%)		82 (25.0%)	54 (65.9%)	28 (34.1%)	
Degree of differentiation								
I+II	117 (60.6%)	86 (73.5%)	31 (26.5%)	.0141*	213 (64.7%)	117 (54.9%)	96 (45.1%)	.0011**
III	76 (39.4%)	67 (88.2%)	9 (11.8%)		116 (35.3%)	85 (73.3%)	31 (26.7%)	
Lymph node metastasis								
Yes	72 (39.8%)	57 (79.2%)	15 (20.8%)	.9655^ns^	123 (39.9%)	80 (65.0%)	43 (35.0%)	.1736^ns^
No	109 (60.2%)	86 (78.9%)	23 (21.1%)		185 (60.1%)	106 (57.3%)	79 (42.7%)	
Clinical stage								
I+II	136 (73.9%)	105 (77.2%)	31 (22.8%)	.5592^ns^	239 (75.4%)	140 (58.6%)	99 (41.4%)	.2043^ns^
III+IV	48 (26.1%)	39 (81.3%)	9 (18.8%)		78 (24.6%)	52 (66.7%)	26 (33.3%)	

Abbreviations: FGFR1, fibroblast growth factor receptor 1; FISH, fluorescence in situ hybridization; IHC, immunohistochemistry.

*P* values are calculated according to chi‐square test. ^ns^ *P* > .05, *P ≤ .05, and **P ≤ .01. FISH analysis was run onto SQCLC and SCLC samples only.

**TABLE 3:** FGFR1 amplification and protein expression in SQCLC patient samples

Feature	FISH				IHC			
	Cases	−	+	*P*	Cases	−	+	*P*
Gender								
Male	134 (85.9%)	99 (73.9%)	35 (26.1%)	.0818^ns^	144 (84.2%)	103 (71.5%)	41 (28.5%)	.372^ns^
Female	22 (14.1%)	20 (90.9%)	2 (9.1%)		27 (15.8%)	17 (63.0%)	10 (37.0%)	
Age								
≥60	122 (78.2%)	91 (74.6%)	31 (25.4%)	.3467^ns^	129 (75.4%)	86 (66.7%)	43 (33.3%)	.0788^ns^
<60	34 (21.8%)	28 (82.4%)	6 (17.6%)		42 (24.6%)	34 (81.0%)	8 (19.0%)	
Degree of differentiation								
I+II	117 (75.0%)	86 (73.5%)	31 (26.5%)	.1577^ns^	131 (76.6%)	89 (67.9%)	42 (32.1%)	.2473^ns^
III	39 (25.0%)	33 (84.6%)	6 (15.4%)		40 (23.4%)	31 (77.5%)	9 (22.5%)	
Lymph node metastasis								
Yes	64 (42.1%)	49 (76.6%)	15 (23.4%)	.9182^ns^	71 (42.5%)	57 (80.3%)	14 (19.7%)	.0188*
No	88 (57.9%)	68 (77.3%)	20 (22.7%)		96 (57.5%)	61 (63.5%)	35 (36.5%)	
Clinical stage								
I+II	110 (71.0%)	82 (74.5%)	28 (25.5%)	.4696^ns^	121 (71.2%)	79 (65.3%)	42 (34.7%)	.0172*
III+IV	45 (29.0%)	36 (80.0%)	9 (20.0%)		49 (28.8%)	41 (83.7%)	8 (16.3%)	

Abbreviations: FGFR, fibroblast growth factor receptor 1; FISH, fluorescence in situ hybridization; IHC, immunohistochemistry.

*P* values are calculated according to chi‐square test. ^ns^ *P* > .05, *P ≤ .05, and **P ≤ .01.

## 4 DISCUSSION

High spread and fatality rates of lung cancer lead to an urgent need of finding new molecular‐based treatment options. EGFR and ALK inhibitors are excellent examples of how molecular targeted treatments can improve prognosis and life quality of selected patients with pulmonary AC.^5^, ^6^ However, no comparable therapeutic options have been established in SQCLC so far. *FGFR1* amplification is a frequent alteration in both SQCLC and SCLC with first clinical trials showing durable response in a small fraction of treated patients.^25^

In our study, we investigate correlation between *FGFR1* gene amplification, protein expression, clinicopathological characteristics, and prognosis of lung cancer patients. We used FISH to test for *FGFR1* amplification in 208 SQCLC and 45 SCLC samples. However, we did not screen *FGFR1* gene amplification in AC samples due to its reported scarcity (0%‐3%) (Table S5).^21^, ^26^, ^27^, ^38^ FISH analysis revealed 23% (n = 37) of SQCLC with amplified *FGFR1* gene going along with previous reports (15%‐22%).^17^, ^21^, ^39^ In SCLC, 8% (n = 3) were amplified which was also comparable to literature (5%‐8.7%).^18^, ^38^

Next, we investigated FGFR1 protein expression by IHC in the same patients and in 121 AC patients. As antibody‐based approaches can be perturbed by cross‐reactivity, we validated specificity of the anti‐FGFR1 antibody as well as the staining protocol through staining tonsil and gallbladder tissue samples whose results were comparable to literature.^33^, ^34^ Next, we performed western blot analysis and immunocytochemistry of five different SQCLC cell lines and could confirm FGFR1 expression as described previously.^37^, ^40^ Furthermore, we knocked out *FGFR1* gene in H1703 cell line using CIRSPR/Cas9 system. Deletion of exon 14 caused a frameshift leading to disruption of *FGFR1* expression and loss of ICC signal.

Using the validated staining protocol, we found FGFR1 strongly expressed in 9% of SQCLC, which was comparable to what was published previously (10%).^41^ In AC, we found strong expression in 35% of samples compared to 13% published previously.^41^ In SCLC, we detected strong staining in 4% of samples, which was lower than the 7.2%‐43.7% previously reported.^32^, ^41^, ^42^ Different reasons could explain difference in prevalence of strong FGFR1 expression like the antibody used for staining, cutoff value for strong expression, and patients' characteristics.

*FGFR1* gene amplification and protein levels were significantly higher in patients with differentiation levels of I and II compared to level III (*P* = .001). In SQCLC, high FGFR1 protein expression but not amplification was correlated to early clinical stages and to the absence of lymph node metastasis (*P* = .017 and .019, respectively). Interestingly, our cohort showed a significant correlation between male gender and *FGFR1* amplification (*P* = .027), which supports previous findings.^20^ Conversely, we found that strong protein expression was significantly more common in females (*P* = .019). Furthermore, we compared *FGFR1* amplification and expression to overall survival of patients and could not find a significant association in any of the tested histological lung cancer entities. These results promote previous reports, which have shown no correlation between *FGFR1* amplification or protein expression and overall survival^2^, ^41^, ^43^ over reports that have shown a significant correlation.^21^, ^38^, ^42^

Clinical trials have tested *FGFR1* as a molecular target in SQCLC patients using *FGFR1* amplification as selection biomarker. Modest response rates evolved from these trials led to uncertainty if FGFR1 amplification is the most accurate criteria for patient selection.^25^, ^44^ One explanation of the results is that kinase inhibitors might only be effective if *FGFR1* is expressed and activated, hence an arising question is how *FGFR1* amplification and overexpression are associated. This correlation is interesting knowing that Her2 target in breast cancer, in some cases, has shown no correlation between overexpression and amplification.^45^, ^46^ Previous studies have shown controversial findings regarding association between *FGFR1* amplification and mRNA/protein expression.^20^, ^47^, ^48^ In this regard, our analysis of 129 SQCLC and 36 SCLC patients revealed no significant correlation between *FGFR1* amplification and protein expression (*P* = .54 in SQCLC and *P* = .99 in SCLC). In like manner, we observed high expression of FGFR1 protein in the SQCLC cell line LK‐2 where *FGFR1* gene is not amplified. Interestingly, LK‐2 cell line showed high sensitivity to FGFR1 inhibitors.^37^ These results suggest that FGFR1 expression might be a more accurate biomarker for existence of the molecular target. Considering that IHC is more broadly available and technically easier than FISH analysis, it might be interesting to test FGFR1 overexpression rather than amplification as selection biomarker for FGFR1 inhibition in clinical trials.

In summary, our study demonstrates that neither *FGFR1* amplification nor protein expression correlate to overall survival in SCLC, SQCLC, or AC patients. This is an advantage in clinical studies testing *FGFR1* inhibitors as differences in survival can more easily be assigned to treatment success rather than only prognostic differences in the two groups. Furthermore, while in literature *FGFR1* amplification is very rarely described in AC, we showed strong FGFR1 expression in 35% (n = 40) of cases. In our analysis, we found that *FGFR1* amplification and protein expression do not seem to be directly correlated in SQCLC and SCLC. These findings are important to consider for selecting patients in clinical trials with *FGFR1* inhibitors.

## CONFLICT OF INTEREST

The authors declare that they have no conflict of interest.

## AUTHOR CONTRIBUTIONS

HB conceived and supervised the project. OE, AML, KS, SH, LL, SY, KRJ, and HUS performed experiments. AE, BCD, MH, and PS contributed clinical samples and/or patient characteristics. HB and OE wrote the manuscript with final approval of all authors.

## Supporting information

Supplementary Material

Supplementary Material

## Data Availability

The data that support the findings of this study are available on request from the corresponding author. The data are not publicly available due to privacy or ethical restrictions.
