# Supplementary material for: Fibroblast growth factor receptor 1 gene amplification and protein expression in human lung cancer
Source: Cancer Med. 2020 Mar 24;9(10):3574–83. doi: 10.1002/cam4.2994 (PMC7288860; doi:10.1002/cam4.2994)
Supplement: Supplementary file 2 — Supplementary Material [file CAM4-9-3574-s002.docx]

| **Supplementary Table 2.** FGFR1/CEN8 ratios in different regions among SQCLC patients (60 nuclei each). | | | | | | | | |
| --- | --- | --- | --- | --- | --- | --- | --- | --- |
|  | **Patient 1** | **Patient 2** | **Patient 3** | **Patient 4** | **Patient 5** | **Patient 6** | **Patient 7** | **Patient 8** |
| Region 1 | 2.3 | 3.0 | 1.7 | 4.5 | 3.2 | 2.6 | 4.3 | 4.8 |
| Region 2 | 2.1 | 2.2 | 1.9 | 6.2 | 2.0 | 2.6 | 2.7 | 2.1 |
| Region 3 | 2.2 | 2.5 | 1.6 | 4.6 | 3.2 | 2.6 | 3.0 | 4.5 |
| SD | 0.084 | 0.317 | 0.120 | 0.799 | 0.557 | 0.026 | 0.680 | 1.183 |
| Variance | 0.007 | 0.100 | 0.014 | 0.639 | 0.311 | 0.001 | 0.463 | 1.398 |
| FGFR1: fibroblast growth factor receptor 1; SQCLC: Squamous cell lung cancer | | | | | | | | |

| **Supplementary Table 3** FGFR1 protein expression in AC patient samples | | | | |
| --- | --- | --- | --- | --- |
| **Feature** | **IHC** | | | |
| Gender | Cases | - | + | p |
| Male | 62 | 23 (37.1%) | 39 (62.9%) | 0.881 |
| Female | 52 | 20 (38.5%) | 32 (61.5%) | ns |
| Age |  |  |  |  |
| ≥60 | 87 | 33 (37.9%) | 54 (62.1%) | 0.9333 |
| <60 | 27 | 10 (37.0%) | 17 (63.0%) | ns |
| Degree of differentiation | |  |  |  |
| I+II | 82 | 28 (34.1%) | 54 (65.9%) | 0.2077 |
| III | 32 | 15 (46.9%) | 17 (53.1%) | ns |
| Lymph node metastasis | |  |  |  |
| Yes | 40 | 11 (27.5%) | 29 (72.5%) | 0.213 |
| No | 66 | 26 (39.4%) | 40 (60.6%) | ns |
| Clinical stage | |  |  |  |
| I+II | 86 | 33 (38.4%) | 53 (61.6%) | 0.4807 |
| III+IV | 26 | 8 (30.8%) | 18 (69.2%) | ns |
| FGFR: fibroblast growth factor receptor 1, IHC: immunohistochemistry. p values are calculated according to Chi-Square test. ns = p>0.05, * = p ≤0.05 and ** = p ≤0.01. | | | | |

| **Supplementary Table 4** FGFR1 amplification and protein expression in SCLC patient samples | | | | | | | | | |
| --- | --- | --- | --- | --- | --- | --- | --- | --- | --- |
| **Feature** | **FISH** | | | | **IHC** | | | | |
| Gender | Cases | - | + | p | Cases | - | + | p |  |
| Male | 27 | 24 (88.9%) | 3 (11.1%) | 0.2715 | 33 | 30 (90.9%) | 3 (9.1%) | 0.4106 |  |
| Female | 10 | 10 (100.0%) | 0 (0.0%) | ns | 11 | 9 (81.8%) | 2 (18.2%) | ns |  |
| Age |  |  |  |  |  |  |  |  |  |
| ≥60 | 27 | 24 (88.9%) | 3 (11.1%) | 0.2715 | 30 | 28 (93.3%) | 2 (6.7%) | 0.1231 |  |
| <60 | 10 | 10 (100.0%) | 0 (0.0%) | ns | 13 | 10 (76.9%) | 3 (23.1%) | ns |  |
| Degree of differentiation | |  |  |  |  |  |  |  |  |
| I+II | 0 | - | - | - | 0 | - | - | - |  |
| III | 37 | 34 (91.1%) | 3 (8.1%) |  | 44 | 39 (88.6%) | 5 (11.4%) |  |  |
| Lymph node metastasis | |  |  |  |  |  |  |  |  |
| Yes | 8 | 8 (100.0%) | 0 (0.0%) | 0.2589 | 12 | 12 (100.0%) | 0 (0.0%) | 0.1248 |  |
| No | 21 | 18 (85.7%) | 3 (14.3%) | ns | 23 | 19 (82.6%) | 4 (17.4%) | ns |  |
| Clinical stage | |  |  |  |  |  |  |  |  |
| I+II | 26 | 23 (88.5%) | 3 (11.5%) | 0.5344 | 32 | 28 (87.5%) | 4 (12.5%) | 0.5153 |  |
| III+IV | 3 | 3 (100.0%) | 0 (0.0%) | ns | 3 | 3 (100.0%) | 0 (0.0%) | ns |  |
| FGFR: fibroblast growth factor receptor 1, FISH: Fluorescence in-situ hybridization and IHC: immunohistochemistry. P values are calculated according to Chi-Square test. ns = p>0.05, * = p ≤0.05 and ** = p ≤0.01. | | | | | | | | |  |

| **Supplementary Table 5.** Review of FGFR1 gene amplification in AC of the lung | | | | |
| --- | --- | --- | --- | --- |
| **Order** | **Author** | **Method** | **Sample size** | **Prevalence** |
| 1 | Russel et al. (2014) | FISH | 99 | 0% |
| 2 | Schildhaus et al. (2012) | FISH | 97 | 0% |
| 3 | Weiss et al. (2010) | FISH | 77 | 1.3% |
| 4 | Cihoric et al. (2014) | FISH | 137 | 2.2% |
| 5 | Seo et al. (2014) | FISH | 230 | 3% |
| 6 | Dutt et al. (2011) | SNP array | 588 | 3% |
| FGFR1: fibroblast growth factor receptor 1; AC: Adenocarcinoma | | | | |

**Supplementary Figure 1**


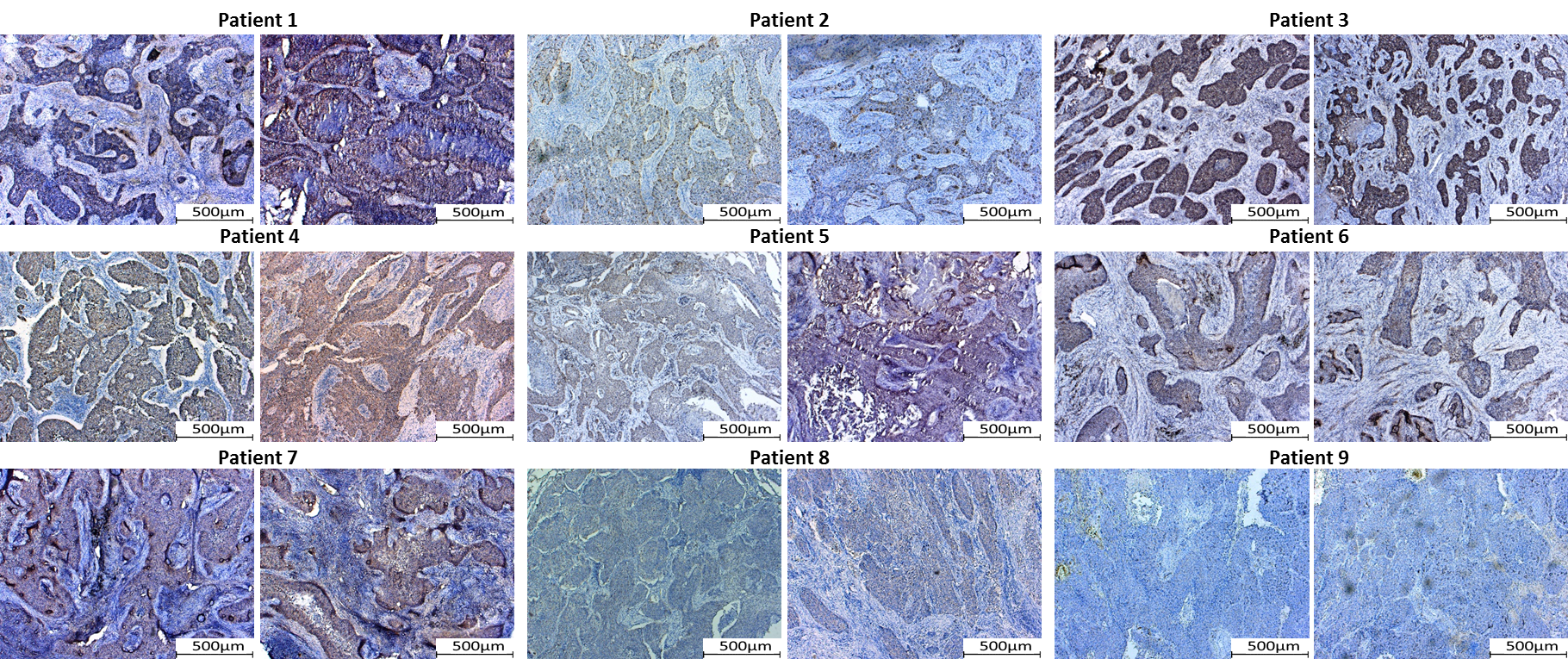


**Supplementary Figure 3: Homogenous expression of FGFR1 among a sample of nine patients.** Figures were taken at a low magnification of 4X.

**Supplementary Figure 2**


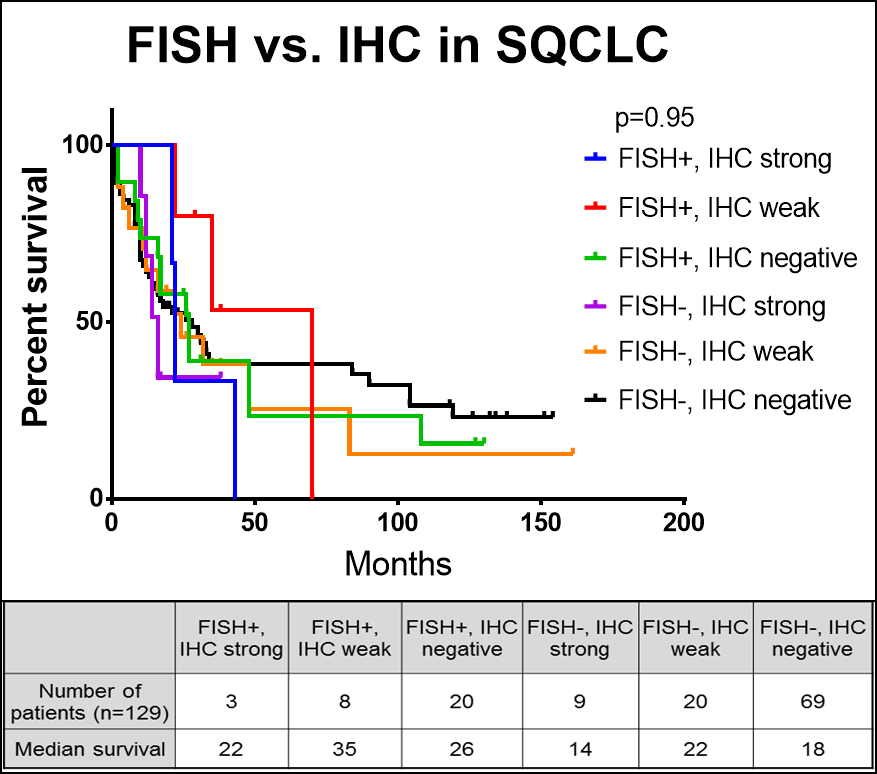


**Supplementary Figure 1: Kaplan-Meier curves correlating patients overall survivals between different groups of SQCLC patients based on FGFR1 amplification and expression status**. P-values were calculated according to Chi-square test.

**Supplementary Figure 3**

**Supplementary Figure 2: Correlation between FGFR1 gene amplification and protein expression in SCLC samples**. P-values were calculated according to Chi-square test.
